# Patterns of DNA Barcode Variation in Canadian Marine Molluscs

**DOI:** 10.1371/journal.pone.0095003

**Published:** 2014-04-17

**Authors:** Kara K.S. Layton, André L. Martel, Paul DN. Hebert

**Affiliations:** 1 Biodiversity Institute of Ontario, University of Guelph, Guelph, Ontario, Canada; 2 Research and Collections (Zoology), Canadian Museum of Nature, Gatineau, Quebec, Canada; Australian Museum, Australia

## Abstract

**Background:**

Molluscs are the most diverse marine phylum and this high diversity has resulted in considerable taxonomic problems. Because the number of species in Canadian oceans remains uncertain, there is a need to incorporate molecular methods into species identifications. A 648 base pair segment of the cytochrome *c* oxidase subunit I gene has proven useful for the identification and discovery of species in many animal lineages. While the utility of DNA barcoding in molluscs has been demonstrated in other studies, this is the first effort to construct a DNA barcode registry for marine molluscs across such a large geographic area.

**Methodology/Principal Findings:**

This study examines patterns of DNA barcode variation in 227 species of Canadian marine molluscs. Intraspecific sequence divergences ranged from 0–26.4% and a barcode gap existed for most taxa. Eleven cases of relatively deep (>2%) intraspecific divergence were detected, suggesting the possible presence of overlooked species. Structural variation was detected in COI with indels found in 37 species, mostly bivalves. Some indels were present in divergent lineages, primarily in the region of the first external loop, suggesting certain areas are hotspots for change. Lastly, mean GC content varied substantially among orders (24.5%–46.5%), and showed a significant positive correlation with nearest neighbour distances.

**Conclusions/Significance:**

DNA barcoding is an effective tool for the identification of Canadian marine molluscs and for revealing possible cases of overlooked species. Some species with deep intraspecific divergence showed a biogeographic partition between lineages on the Atlantic, Arctic and Pacific coasts, suggesting the role of Pleistocene glaciations in the subdivision of their populations. Indels were prevalent in the barcode region of the COI gene in bivalves and gastropods. This study highlights the efficacy of DNA barcoding for providing insights into sequence variation across a broad taxonomic group on a large geographic scale.

## Introduction

DNA barcoding employs sequence diversity in a 648 base pair region of the cytochrome *c* oxidase subunit I (COI) gene to distinguish species [1]–[3]. Past work has shown that sequence divergences are generally much greater between than within species [1]. Because of this fact, DNA barcoding aids both the identification of known species and the discovery of overlooked taxa [4]. The latter application has revealed that the incidence of sibling species is often high enough to lead to serious inaccuracies in estimates of biodiversity [3], [5]. In light of this, it is increasingly recognized that molecular approaches need to be incorporated into biodiversity surveys. Although marine molluscs have been the subject of considerable research, the number of species in Canadian waters remains uncertain with estimates ranging from 700 to 1200. This uncertainty reflects taxonomic problems linked to the fact that molluscs are the most diverse phylum of marine life, with more than 50,000 described species, coupled with a shortage of taxonomists [6]. In addition, molluscs exhibit complex larval stages, frequently have cryptic taxa, and substantial phenotypic plasticity, all factors that impede morphological approaches to species identification [7], [8]. Because morphological analysis confronts so many challenges, it is imperative to integrate molecular diagnostics into the identification of molluscs.

Several prior studies have validated the efficacy of DNA barcoding in the discrimination of mollusc species, but most of this work has targeted a particular family or order. For instance, a detailed study of barcode diversity in cowries (Cypraeidae) demonstrated the general effectiveness of the approach, but showed that a fixed sequence threshold could not be used for species diagnosis [9]. However, this study did indicate that DNA barcoding was a powerful aid to the identification of cowries when paired with strong taxonomic validation and comprehensive sampling [9]. More recent studies have extended these results by establishing the value of DNA barcoding in resolving cryptic species complexes in several molluscan families [10]–[12] and delineating species of Chinese neogastropods [13].

Despite the demonstrated utility of DNA barcoding in molluscs, no study has aimed to assemble a comprehensive barcode registry for the mollusc fauna of a large geographic region. The present investigation addresses this deficit, beginning the construction of a DNA barcode reference library for Canadian marine molluscs. This study also investigates variation in nucleotide composition among molluscs, and its impact on levels of genetic divergence. Finally, patterns of insertion and deletion in the barcode region are analyzed.

## Methods

### Ethics statement

Field work in Churchill, Manitoba was conducted under permits issued by Manitoba Conservation Wildlife and Ecosystem Protection to the Churchill Northern Studies Centre (CNSC) for research in the Churchill Wildlife Management Area. Collections in Alaska were conducted under a fish resource permit granted to Sarah Hardy by the State of Alaska Department of Fish and Game for scientific/educational purposes. Collections in New Brunswick were conducted under a permit from Fisheries and Oceans Canada. No specific permits were required for other collection activities as they were not conducted on privately owned or protected land. No field studies involved the collection of endangered or protected species.

### Specimen collection and identification

**Figure 1:**
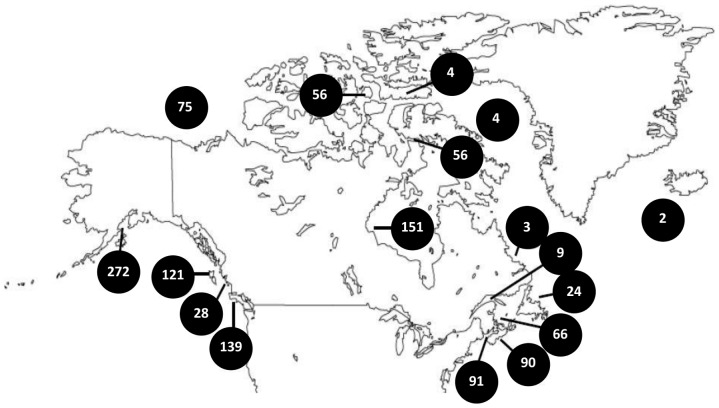
Collection sites. Sampling locations and the number of specimens examined in this study. Only specimens that generated a sequence are shown. Twenty-three sequences obtained from GenBank are not included as they lack locality information.

A total of 2352 specimens were collected from 1999 to 2012 at sites across Canada (Figure 1). One quarter (666) of these specimens were collected from Alaska, Greenland and Iceland, although species found in these locations also exist in Canadian waters. Specimen details, sequences, and trace files are available from the Dataset at dx.doi.org/10.5883/DS-COIMOL on BOLD (Barcode of Life Data Systems) [14], while the specimens are held at the Biodiversity Institute of Ontario. Sequences have also been deposited in GenBank (Accessions AB084110, AF120639, AF120640, AY260813 - AY260818, AY260821, AY260822, AY260824 - AY260833, AY342055, DQ093531, GU802389 - GU802397, GU802411, GU802415 - GU802432, HM431980, HM432253, HM862494, HM862496, HM884235 - HM884236, HM884239 - HM884242, HM884246 - HM884248, HM884251 - HM884255, HM888433, HQ558792, HQ919139 - HQ919140, HQ919142, HQ919168, HQ919183 - HQ919186, HQ919194, HQ919200, JF862383 - JF862384, JF862386 - JF862388, JF884198 - JF884199, JN802379 - JN802388, JN802503 - JN802511, KF643244 - KF643466, KF643468 - KF644349, NC_005840, NC_006162). When possible, five specimens per species were collected from intertidal or subtidal habitats using plankton nets, small dredges, and SCUBA diving, but samples from the Beaufort Sea were collected from deep subtidal soft-bottom habitats using an Agassiz trawl. Specimens were immediately fixed in 90–100% ethanol, with subsequent replacement of ethanol to prevent its dilution. During fixation, the opercula of gastropods were removed, and the shells of bivalves were separated to ensure preservation of internal tissues. After each collecting trip, specimens were placed in fresh 95% ethanol and stored at −20°C. When possible, specimens were identified to a species-level based on comparisons with reference specimens in the mollusc collection at the Canadian Museum of Nature with name usage following the World Register of Marine Species (WoRMS). Approximately 8% of the specimens could not be identified to a species-level because they were immature, but most of these were assigned to a genus and to an interim species. Twenty five scaphopod sequences from species known to occur in Canada were also mined from GenBank to increase the number of representatives in this class (Accessions AB084110, AF120639, AF120640, AY260813 - AY260818, AY260821, AY260822, AY260824 - AY260833, AY342055, DQ093531, NC_005840, NC_006162).

### DNA extraction, amplification and sequencing

DNA extracts were prepared from a small sample of muscle tissue from each specimen. Tissue samples were placed in cetyltrimethylammonium bromide (CTAB) lysis buffer solution with proteinase K and incubated for 12 hours at 56°C. DNA was then extracted using a manual glass fibre plate method [15]. After incubation, the DNA was eluted with 40 µl of ddH_2_O. After resuspension, 2 µl of each DNA extract was placed into a well in another plate and 18 µl of ddH_2_O was added to dilute salts or mucopolysaccharides that might inhibit PCR. Three primer sets were employed to maximize amplicon recovery (dgLCO1490/dgHCO2198, LCO1490_t1/HCO2198_t1 and BivF4_t1/BivR1_t1). The primer set that generated an amplicon for a particular specimen, and the primer sequences are recorded on BOLD. A primer cocktail (C_LepFolF/C_LepFolR) was used in a second round of PCR for specimens that failed to amplify in the first trial. Each PCR reaction included 2 µl of diluted DNA, 6.25 µl 10% trehalose, 2 µl ddH_2_0, 1.25 µl 10× PCR buffer, 0.625 µl MgCl_2_ (50 mM), 0.125 µl of each forward and reverse primer (10 µM), 0.0625 µl dNTP (10 mM) and 0.06 µl Platinum Taq polymerase, producing a total reaction volume of 12.5 µl. The thermocycling regime consisted of one cycle of 1 min at 94°C, 40 cycles of 40 s at 94°C, 40 s at 52°C, and 1 min at 72°C, and finally 5 min at 72°C. We increased the number of PCR cycles to 40 to compensate for a lower DNA template concentration. E-Gels (Invitrogen) were used to screen for amplification success and all positive reactions were bidirectionally sequenced using BigDye v3.1 on an ABI 3730xl DNA Analyzer (Applied Biosystems). Sequences were manually edited using CodonCode Aligner (CodonCode Corporation) and an amino acid alignment was generated by eye in MEGA5 [16]. MEGA5 was also used to assess the prevalence and location of insertions and deletions (indels) before they were mapped onto the secondary structure of COI using the BarcodeStructureMap Python script ver 0.9 (Schonfeld, unpublished). Sequences containing more than 1% ambiguities, stop codons, double peaks or that were shorter than 220 bp were removed from further analysis. Sequencing success was assessed using a Pearson's Chi-Square test to determine if there were significant differences in sequence recovery among the classes of molluscs examined in this study.

### Data analysis

A Kimura-2-parameter (K2P) distance model was employed in MEGA5 to construct a neighbour-joining (NJ) tree which served as a preliminary basis for species recognition [16], [17]. Genetic distances, including intra- and interspecific divergence along with nearest neighbour distance, were calculated with the K2P distance model [17], and overall data were compared using the ‘Distance Summary’ and ‘Barcode Gap Analysis’ tools on BOLD [14]. Maximum intraspecific divergence was plotted against nearest neighbour distance to determine how often nearest neighbour distances were greater than intraspecific divergences, indicating the presence of a barcode gap. In addition, the ‘Sequence Composition’ tool on BOLD was used to examine variation in GC content among species in the 35 orders analyzed [14]. Species numbers were determined by two approaches: i) morphology and ii) through the number of COI sequence clusters. The latter approach employed three algorithms designed for cluster recognition - Barcode Index Number (BIN) [18], Automated Barcode Gap Discovery (ABGD) [19] and jMOTU [20]. The BIN algorithm only analyzed sequences greater than 500 bp in length, while the other two algorithms examined all sequences greater than 400 bp. For jMOTU, a BLAST identity filter of 99 was used along with a sequence alignment overlap of 60% of the minimum sequence length. Clusters were delineated based on a 2% threshold, meaning that 13 base pair differences were required for OTU recognition. For ABGD, values of Pmin and Pmax were set to 0.0006 and 0.17 respectively.

The Picante and VEGAN packages in Revolution R were used to perform linear regressions to determine if the number of individuals sampled within a species impacted estimates of intraspecific divergence and similarly if the number of species sampled from a genus impacted mean nearest neighbour distances [21], [22]. Nearest neighbour distances were then plotted against sampling completeness categories (%) to determine how variation in sampling within genera affected mean nearest neighbour distances, and an ANOVA was used to determine statistical significance. The World Register of Marine Species (WoRMS) was used to determine how many species are currently known from each genus. A chi-square test of homogeneity (Revolution R) was used to determine whether nucleotide frequencies were homogeneous among classes; P-values less than 0.05 were considered as significant. Species with intraspecific divergences greater than 2% were treated as potential cryptic complexes and neighbour-joining trees were created in MEGA5. Three scaphopod species mined from GenBank were excluded from this analysis due to possible misidentifications. Divergence times were estimated in MEGA5 assuming a substitution rate of 2% per million years [16], [23]–[24]. Lastly, the boot and Hmisc packages in Revolution R were used to test whether mean nearest neighbour distance was correlated with mean GC content across 138 molluscan genera [25].

## Results

### Sequence recovery

A total of 1214 COI sequences were recovered from the 2352 specimens. The LCO1490_t1 and HCO2198_t1 primer set, along with a 1∶10 dilution of DNA and an annealing temperature of 52°C, generated the highest success in sequence recovery. Success rates showed significant variation among classes, ranging from 82.8% in polyplacophorans to 42.5% in gastropods and 33.1% in bivalves. Reflecting the fact that some DNA was degraded and the need to use internal primers in these cases, sequences ranged in length from 268 to 658 bp, but 89% were greater than 600 bp. Values of intraspecific divergence ranged from 0% to 26.4%, while divergences between congeners ranged from 0.3% to 58.4% (Table S1).

### COI variation in marine molluscs

**Figure 2:**
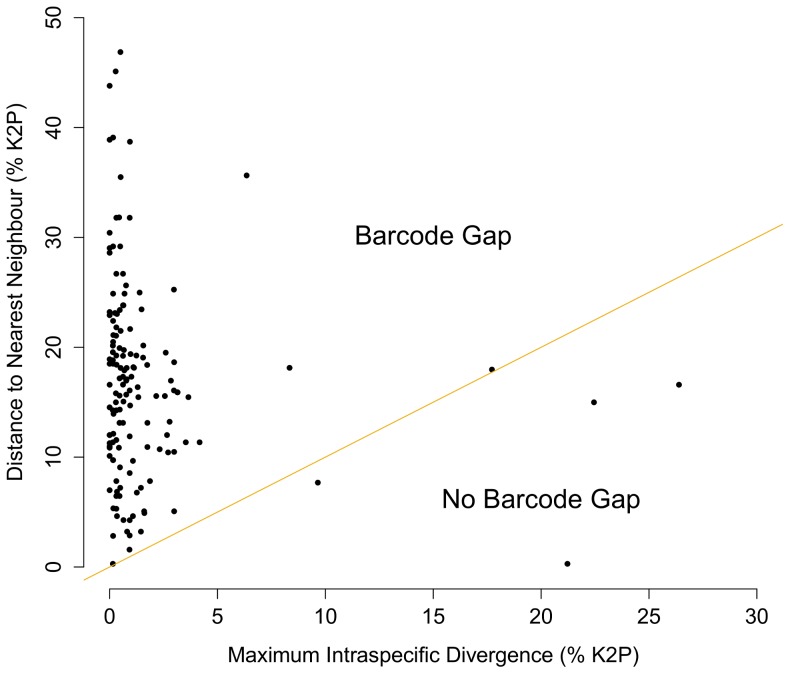
The barcode gap. Maximum intraspecific divergence (% K2P) in the barcode region of cytochrome *c* oxidase subunit I plotted against nearest neighbour distance (% K2P) for the 227 morphospecies examined in this study. Points above the line indicate species with a barcode gap.

Morphological study indicated the presence of 227 species; 80 were represented by a single specimen, while the other 147 species had an average of 8 specimens (range 2–56) (Table S1; Table S2). All species had one or more sequence records >400 bp in length. No barcode sharing was detected among individuals of different species and a barcode gap was present for all but six cases (Figure 2). Each of these exceptions was the result of deep intraspecific divergence, likely arising from the presence of cryptic species complexes (Figure 2). Algorithms for OTU determination generated estimates of 235 (BIN), 247 (jMOTU) and 250 (ABGD). Because 53 specimens representing 16 morphologically identified species lacked BIN assignments (because their sequence records were <500 bp) a more accurate BIN estimate may be upwards of 250, suggesting the congruence in cluster count among the three algorithms is strong. The BIN, jMOTU and ABGD algorithms generated 89, 98 and 100 singletons, respectively. The ABGD algorithm generated nine potential clustering schemes and scheme five was chosen as most appropriate.

**Figure 3:**
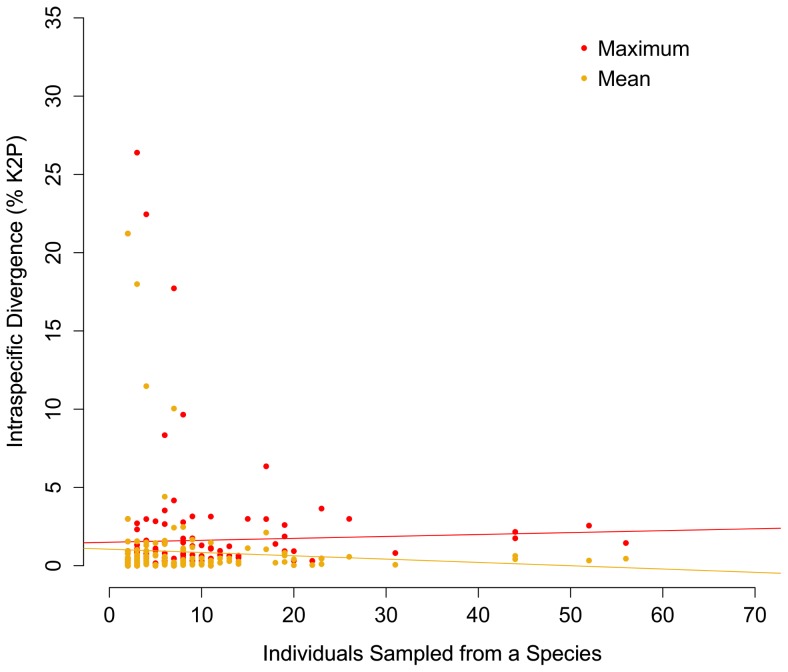
The relationship between COI distance and sample size within species. Maximum and mean intraspecific divergences (% K2P) at COI plotted against the number of individuals analyzed for 147 species of Canadian marine molluscs. The regression between sample size and mean divergence is insignificant (P = 0.38; R^2^ = 0.005) as well as the regression between sample size and maximum divergence (P = 0.72; R^2^ = 0.0009).

**Figure 4:**
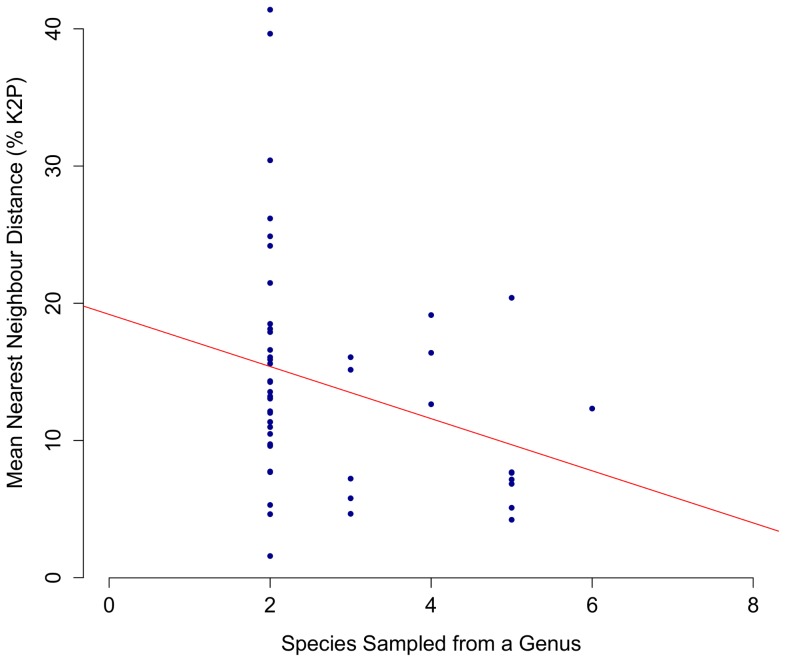
The relationship between COI distance and sample size within genera. Mean nearest neighbour distance (% K2P) at COI plotted against the number of species sampled from each genus of marine mollusc with ≥2 species (N = 50). The regression was insignificant (P = 0.052; R^2^ = 0.08). Morphospecies lacking a generic identification were excluded from analysis.

**Figure 5:**
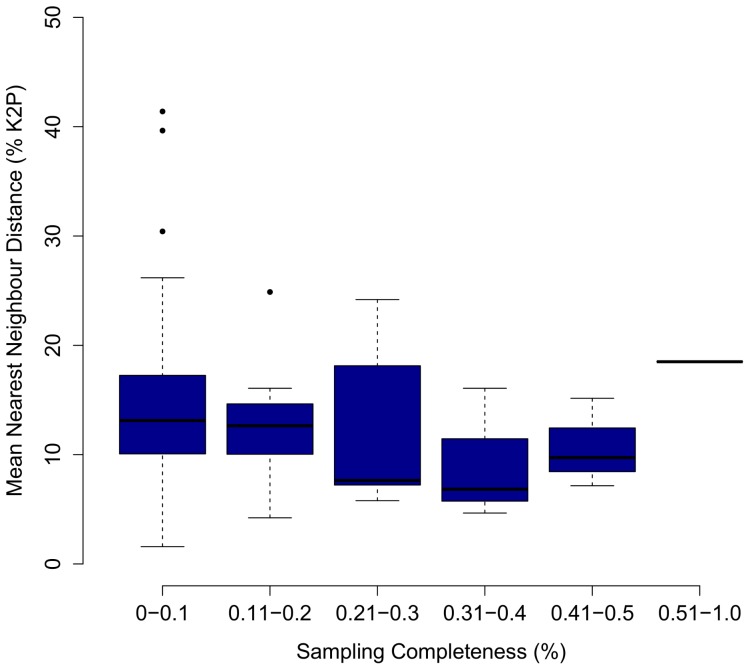
The relationship between COI distance and sampling completeness within genera. Mean nearest neighbour distance (% K2P) at COI plotted against sampling completeness (%) of each genus of marine mollusc with ≥2 species (N = 50). The ANOVA was insignificant (P = 0.77). Morphospecies lacking a generic identification were excluded from analysis.

**Figure 6:**
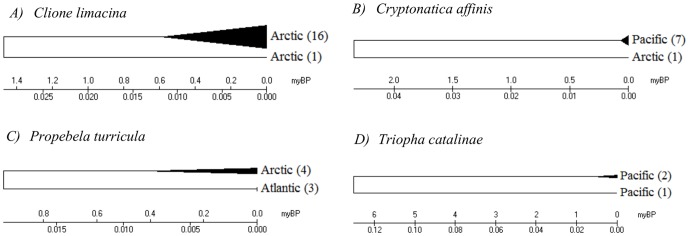
Deep divergences at COI within species of gastropod. Neighbour-joining trees (K2P) with locality information for four gastropod species showing more than 2% sequence divergence at COI. Triangles on the NJ tree represent compressed clades, with sample size provided in brackets.

**Figure 7:**
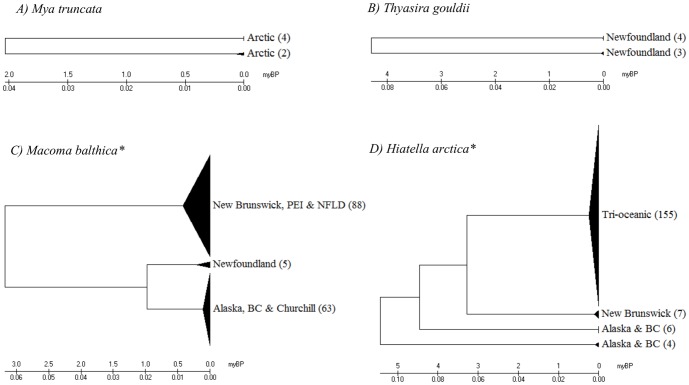
Deep divergences at COI within species of bivalve. Neighbour-joining trees (K2P) with locality information for four bivalve species showing more than 2% sequence divergence at COI. Triangles on the NJ tree represent compressed clades, with sample size provided in brackets. *(Layton 2012)

Maximum and mean intraspecific divergence did not significantly differ with the number of individuals analyzed per species (Figure 3; P = 0.72, P = 0.38). Mean nearest neighbour distance appeared to decrease with the number of species analyzed from a genus, but the regression was not significant (Figure 4; P = 0.052). However, mean nearest neighbour distance did not significantly differ with sampling completeness within genera (Figure 5; P = 0.77). Eleven species demonstrated intraspecific divergences greater than 2%. Neighbour-joining trees (K2P) and locality information are provided for eight of these cases (data for *Hiatella arctica* and *Macoma balthica* derive from Layton 2012) (Figure 6; Figure 7).

### Variation in nucleotide composition

**Figure 8:**
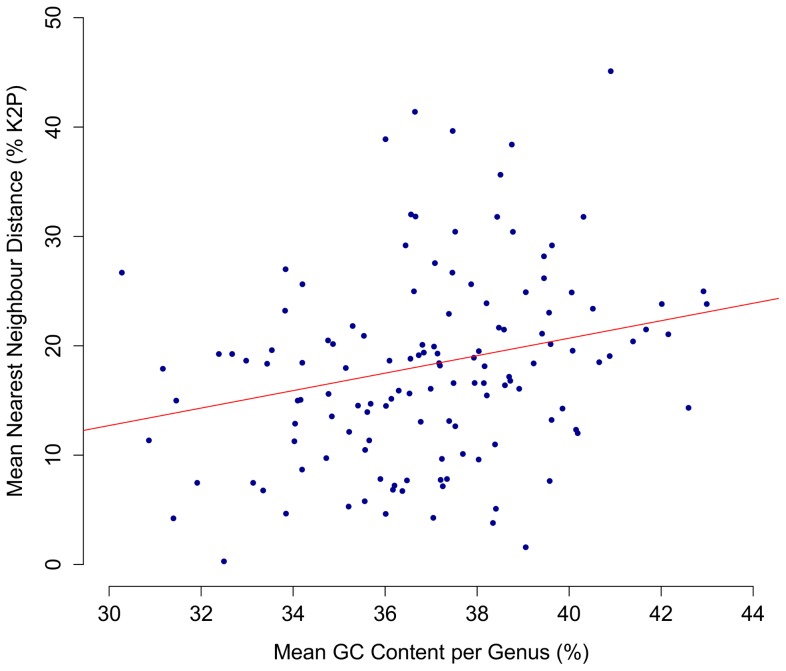
The relationship between GC content and genetic distance across genera. Mean nearest neighbour distance (% K2P) at COI plotted against mean GC content (%) for congeneric species in the 138 genera of molluscs analyzed in this study. The correlation was significant (P<0.001; R = 0.29).

Mean GC content averaged 36.9%, but showed considerable variation (range 24.5%–46.5%). A chi-square test of homogeneity demonstrated significant variation in nucleotide frequencies among species in each of five molluscan classes (P<0.001). Mean nearest neighbour distances between congeneric species showed a significant (P<0.001) positive correlation with mean GC content (Figure 8).

### Distribution of indels

Indels were only detected in two of the five classes, Bivalvia and Gastropoda, occurring in nearly half (49%) of the bivalve species versus just 9% of the gastropods. Indels were detected in 37 bivalve species from 11 families, and in 11 gastropod species from four families. All indels were in multiples of three nucleotides, suggesting they did not derive from pseudogenes. They were conserved in seven bivalve families, but varied between genera in two families and between species in two families. A single codon insertion occurred in all myids and in the single arcid and glycymerid species. Moreover, there was a three codon insertion in all thyasirids, a one codon deletion in all tellinids, a three codon deletion in both ostreids, and a two codon deletion in the single propeamussid species. Other families showed variation. For example, a single codon deletion was observed in *Cyclocardia borealis*, but *Cyclocardia crassidens* possessed a single codon insertion, a three codon deletion and another single codon deletion. While both astartids (*Astarte montagui*, *Astarte borealis*) shared a single codon insertion and a three codon deletion, *A. borealis* had an additional one codon insertion. One codon deletion was observed in *Mactromeris polynyma*, while other mactrids had no indels. Lastly, while all *Mytilus*, *Musculus* and *Crenella* species had a single codon insertion, another mytilid genus (*Modiolus*) lacked it. Indels were conserved in three of the four gastropod families, but varied between lottiid genera as all five *Lottia* species shared a single codon insertion that was absent in *Discurria insessa*. Conversely, all pyramidellids (*Boonea cf. bisuturalis*, *Odostomia sp.KL01*, *Odostomia sp. KL0*2) and onchidiids (*Onchidella borealis*, *Onchidella cf. carpenteri*) had a one codon deletion, while the sole limacinid member (*Limacina helicina*) had a three codon deletion and an additional one codon deletion.

**Figure 9:**
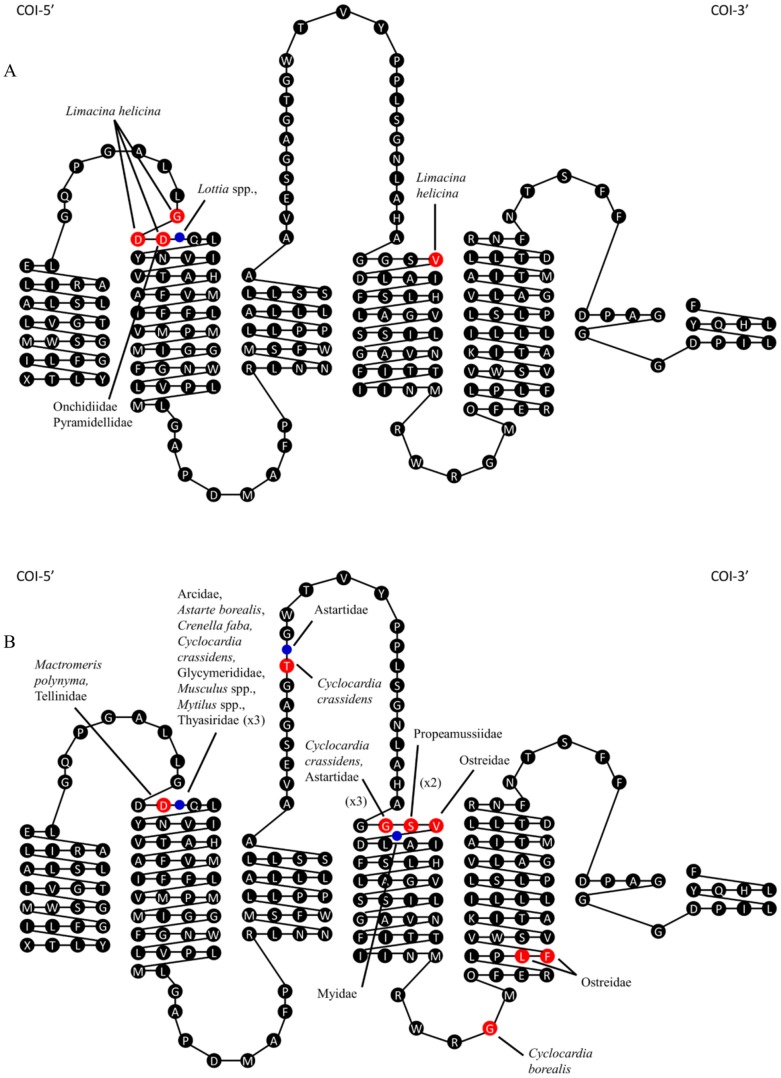
Mapping insertions and deletions in COI. Secondary structure of COI marked with insertions and deletions A) gastropods and B) bivalves. Insertions are marked with a blue circle, while deletions are marked with a red circle.

When indels were mapped onto the secondary structure of COI, it was apparent that most were close to the external loops, primarily the first (Figure 9). Fifteen species from five bivalve families (Arcidae, Astartidae, Carditidae, Glycymerididae, Mytilidae) and one gastropod family (Lottiidae) shared a single codon insertion at site 37 in the alignment. All thyasirids also had a three codon insertion at the same site. Moreover, eleven species from two bivalve families (Mactridae, Tellinidae) and two gastropod families (Onchidiidae, Pyramidellidae) shared a single codon deletion event at site 36 in the alignment. Additionally, the gastropod *Limacina helicina* and the bivalves *Crassostrea gigas* and *Crassostrea virginica* shared a single codon deletion event at site 132 in the alignment. These results suggest that some sites in the barcode region are particularly susceptible to indels. The largest insertion was three codons in length and spanned sites 37–39 in the thyasirids. The largest deletion was also three codons in length and spanned sites 130–132 in *Cyclocardia crassidens* and the astartids.

## Discussion

### Sequencing success in Mollusca

Although this study employed multiple rounds of PCR, tested different primer cocktails, and modified PCR regimes, only 51.6% of the specimens generated an amplicon. The design of order-level primers would likely enhance success in this group because variation in sequence recovery was detected among classes, with polyplacophorans the highest (82.8%) and bivalves the lowest (33.1%). The variable success in sequence recovery may also reflect variation in mucopolysaccharide levels which often reduce PCR amplification success (Steinke, unpublished). Regardless of its cause, work is needed to improve protocols for DNA extraction and/or PCR amplification in marine molluscs.

### Patterns of sequence variation

The present study has delivered barcode coverage for 227 species of Canadian marine molluscs, nearly 25% of the fauna. The 147 taxa with two or more records showed a mean intraspecific divergence of 0.49%, a value higher than that reported for other marine taxa including polychaetes (0.38%), marine fishes (0.39%), and decapods (0.46%) but lower than echinoderms (0.62%) [3], [26]–[28]. However, when cases of deep sequence divergence (that likely reflect overlooked taxa) were excluded, mean intraspecific divergence dropped to 0.42%. The levels of intraspecific variation detected in this study show general congruence with those reported in other molluscan barcoding studies with mean intraspecific variation typically less than 1% [9], [12]–[13]. By contrast, interspecific divergences were consistently high among taxa [9], [12]–[13]. A barcode gap existed for most species except the few taxa with deep intraspecific divergence.

This study revealed 11 taxa with intraspecific divergences greater than 2% (Figure 6; Figure 7). Prior work has revealed deep mtDNA divergence in some mollusc species, such as the land snail, *Cepaea nemoralis*, where distances reach 12.9% [29]. However, in most other cases, deep divergences are thought to represent different species. For example, the 33.6% divergence between Antarctic and Arctic populations of the pteropod *Limacina helicina* is viewed as evidence for separate species in the two polar regions [30]. Similarly, the Arctic lineage of *Clione limacina* has been described as a separate species from the Antarctic lineage [31], and this study extends this conclusion by suggesting the possible presence of two *Clione* species in the Arctic Ocean with a divergence of 5.9% (Figure 6). Pleistocene glaciations are known to have played an important role in the population subdivision of many Canadian aquatic organisms, producing differentiation between lineages on the Atlantic, Arctic and Pacific coasts [32]–[35]. During glacial cycles, the repeated opening and closing of the Bering Strait caused periods of isolation followed by the exchange of species between Pacific and Atlantic coasts [36]–[38]. Four cases of deep divergence detected in this study (*Cryptonatica affinis, Hiatella arctica, Macoma balthica, Propebela turricula*) show a biogeographic partition between their component lineages. Estimated divergence times for these four cryptic cases all exceed 900,000 years, suggesting their origin through repeated isolation in different coastal glacial refugia (Figure 6; Figure 7). However, doubly uniparental inheritance (DUI) of mtDNA in bivalve lineages can lead to deep sequence divergences between the two sexes [39]–[41]. This phenomenon has been observed in five marine bivalve families, including; Donacidae, Mytilidae, Nuculanidae, Solenidae and Veneridae [42]–[45]. The male genome is most often expressed in gonadal tissue, but some studies have found both male and female mtDNA in the somatic tissue of male *Mytilus*, potentially causing deep splits in species affected by this phenomenon [46], [47]. However, it is important to note that some of the cases of deep divergence detected in this study occurred in species unaffected by DUI. Other cases, such as the two clusters of *Mya truncata* detected in this study, may reflect the inclusion of cryptic taxa known from other sites in the Arctic Ocean [48]. In any case, deep intraspecific divergences often flag overlooked species [4]. For instance, DNA barcoding revealed five cryptic species complexes in the Lepetodrilidae, a family of limpets inhabiting deep-sea hydrothermal vents [11]. Similarly, COI analysis established that the cold-seep bivalve species, *Acesta bullisi*, was actually two species [49]. DNA barcoding has provided similar evidence of overlooked diversity in numerous marine taxa, including fishes and asteroids [50], [51], highlighting the need for integrating molecular approaches into species identifications.

### Insertions and deletions in COI

Prior work has established that indels are usually rare in the barcode region of COI [1], [52]. Most of the molluscan classes examined in this study followed this pattern, but indels were detected in gastropods and especially in bivalves. Most of these indels were positioned in sequence regions coding for amino acids placed near loops that extend into the inter-membrane space and certain sites were hotspots for change as indels were detected at the same position in phylogenetically divergent lineages. For example, some members of four bivalve orders possessed a single codon insertion at site 37, while others did not, indicating its recurrent gain or loss at this site. Interestingly, a recent study discovered that *Thyasira* species possessed three or four additional codons in the COI gene [10], a conclusion corroborated by the detection of a three codon insertion at site 37 in all thyasirids examined in this study. The reason for elevated rates of structural change in certain lineages is uncertain, but the single codon insertion in the lottiids (Patellogastropoda), and a three codon deletion and an additional one codon deletion in *Limacina helicina* have been linked to accelerated rates of nucleotide substitution in these groups [53]. Together with prior work, the present study has established that insertions and deletions in the barcode region of the COI gene are relatively common in some classes of molluscs, suggesting that future work should aim to determine the functional significance of this variation as well as its association with rates of molecular evolution.

### Patterns of nucleotide composition

Significant variation in nucleotide composition was detected among the species in each class. Although the effect of compositional shifts on phylogenetic reconstructions has been well recognized, our analysis indicated a positive correlation between GC content and sequence divergence between congeneric taxa. Future work should examine the impact of variation in GC content on sequence divergence between sister taxa in other groups.

## Supporting Information

Table S1 Intraspecific and nearest neighbour distances and the number of individuals sampled for each of the 227 morphospecies in this study. A hyphen will appear in the intraspecific divergence columns for singletons.
(PDF)

Table S2 Species found at each locality in this study. This table excludes 23 GenBank specimens (Scaphopoda) that lack locality information but are known to occur in Canada.
(PDF)
