# Supplementary material for: Patterns of DNA Barcode Variation in Canadian Marine Molluscs
Source: PLoS One. 2014 Apr 17;9(4):e95003. doi: 10.1371/journal.pone.0095003 (PMC3990619; doi:10.1371/journal.pone.0095003)
Supplement: Table S1 — Intraspecific and nearest neighbour distances and the number of individuals sampled for each of the 227 morphospecies in this study. A hyphen will appear in the intraspecific divergence columns for singletons. (PDF) [file pone.0095003.s001.pdf]

Supporting Information Table S1. **Intraspecific and nearest neighbour distances and the number of individuals sampled for each of the 227 morphospecies in this study.** A hyphen will appear in the intraspecific divergence columns for singletons.

Species	Number of Individuals	Mean Intra-specific (% K2P)	Max Intra-specific (% K2P)	NN Distance (% K2P)
Acanthodoris nanaimoensis	1	-	-	11.21
Acanthodoris pilosa	1	-	-	4.27
Acmaea mitra	10	0.15	0.62	23.82
Admete sp. KL01	3	0	0	10.98
Admete viridula	1	-	-	10.98
Aeolidia papillosa	3	0.41	0.62	19.22
Aeolidia sp. KL01	1	-	-	13.12
Aeolidia sp. KL02	9	0.15	0.46	13.12
Alia carinata	9	0.46	1.26	6.77
Amphissa columbiana	3	0.64	0.93	4.26
Amphissa reticulata	1	-	-	5.45
Amphissa versicolor	1	-	-	4.26
Antalis antillarum	2	0.15	0.15	0.29
Antalis dentalis	1	-	-	2.36
Antalis entalis	3	0.19	0.29	15.81
Antalis inaequicostata	1	-	-	2.36
Antalis sp. PR-2003	1	-	-	0.29
Ariadnaria insignis	6	0.15	0.31	7.82
Armina californica	2	0.51	0.51	21.49
Astarte borealis	1	-	-	17.90
Astarte montagui	12	0.19	0.68	17.90
Axinopsida orbiculata	2	0.16	0.16	9.73
Axinopsida serricata	2	0.16	0.16	9.73
Bathyarca pectunculoides	3	0.30	0.49	29.18
Boonea cf. bisuturalis	2	0.31	0.31	21.81
Boreochiton beringensis	1	-	-	3.80
Boreotrophon cepula	1	-	-	7.69
Boreotrophon truncatus	1	-	-	7.69
Brachioteuthis beani	2	0.15	0.15	18.82
Buccinum aleuticum	3	0.32	0.48	9.07
Buccinum hydrophanum	3	0.11	0.17	5.34
Buccinum senshumaruae	1	-	-	5.34
Buccinum sp. KL01	1	-	-	2.87
Buccinum undatum	2	0.93	0.93	2.87
Cadlina luteomarginata	2	0	0	16.59
Calliostoma canaliculatum	1	-	-	14.33
Calliostoma ligatum	10	0.17	0.47	14.33
Ceratostoma foliatum	3	0	0	10.11
Cidarina cidaris	8	0.19	0.47	23.39
Ciliatocardium ciliatum	6	0.22	0.34	23.03
Clinocardium nuttallii	3	0.10	0.16	20.49
Clione limacina	17	2.12	6.35	35.64
Colus stimpsoni	1	-	-	12.88
Crassostrea gigas	4	0.08	0.16	24.88
Crassostrea virginica	9	0.06	0.69	24.88
Crenella faba	20	0.03	0.32	18.41
Crepidula fornicata	11	1.47	3.14	15.90
Crepidula williamsi	9	1.66	3.15	15.90

Crepipatella lingulata	14	0.26	0.64	15.06
Cryptobranchia concentrica	13	0.29	0.64	23.82
Cryptochiton stelleri	1	-	-	15.64
Cryptonatica affinis	8	2.47	9.65	7.68
Cryptonatica aleutica	2	0	0	6.99
Cryptonatica russa	1	-	-	6.99
Cuspidaria glacialis	1	-	-	30.42
Cuspidaria subtorta	2	0	0	30.42
Cuthona columbiana	1	-	-	18.36
Cyanoplax dentiens	4	0.62	0.93	16.07
Cyanoplax fernaldi	17	1.05	2.98	16.07
Cyclocardia borealis	1	-	-	43.70
Cyclocardia crassidens	3	0.11	0.16	39.09
Cylichna alba	2	0.15	0.15	11.35
Cylichna cf. gouldii	6	1.60	3.53	11.35
Dendronotus cf. robustus	1	-	-	13.22
Dendronotus frondosus	8	1.11	2.78	13.22
Dentalium pilsbryi	2	21.22	21.22	0.29
Diaphana minuta	1	-	-	19.60
Diaulula sandiegensis	1	-	-	16.80
Dirona albolineata	2	0.15	0.15	19.55
Discurria insessa	10	0.06	0.31	19.25
Doris montereyensis	5	0.66	0.97	19.38
Dosidicus gigas	19	0.65	2.60	19.51
Ecrobia truncata	11	0.19	0.46	17.17
Ennucula tenuis	18	0.19	1.39	24.98
Ensis directus	1	-	-	30.43
Entalina tetragona	1	-	-	28.18
Enteroctopus dofleini	1	-	-	7.47
Episiphon yamakawai	1	-	-	18.45
Eubranchus rupium	5	0.70	1.10	18.19
Euspira heros	8	0.18	0.31	5.30
Euspira pallida	1	-	-	5.30
Fissidentalium candidum	1	-	-	20.91
Fissurellidea bimaculata	1	-	-	25.07
Flabellina cf. trophina	1	-	-	20.24
Flabellina salmonacea	5	1.45	2.84	16.96
Flabellina sp. KL01	2	0.77	0.77	16.96
Flabellina verrucosa	7	0.07	0.16	22.40
Gadila aberrans	2	2.99	2.99	18.64
Gastropteron pacificum	13	0.47	1.24	19.25
Gemma gemma	9	0.11	0.31	26.69
Glycymeris septentrionalis	2	0.16	0.16	29.18
Graptacme eborea	2	0.29	0.29	14.99
Haminoea virescens	7	0.22	0.46	19.93
Homalopoma baculum	2	0	0	28.60
Homalopoma luridum	1	-	-	23.76
Ilyanassa obsoleta	12	0.47	0.95	14.70
Janolus fuscus	1	-	-	23.89
Katharina tunicata	8	1.01	1.74	18.39
Keenocardium blandum	2	0.16	0.16	18.50
Keenocardium californiense	2	0	0	18.50
Kellia suborbicularis	1	-	-	27.56
Lacuna sp. KL01	3	1.57	2.32	10.72
Lacuna vincta	10	0.48	1.30	16.37
Lepidochitona flectens	3	1.01	1.33	15.46

Lepidozona interstincta	11	0.33	1.08	4.63
Lepidozona radians	4	0.24	0.34	4.63
Leptochiton alascensis	2	0	0	12.01
Leptochiton rugatus	2	0	0	12.01
Leukoma staminea	1	-	-	27.00
Limacina helicina	2	0.94	0.94	38.71
Lirabuccinum dirum	8	0.04	0.18	13.94
Lirularia succincta	2	0.95	0.95	21.66
Littorina littorea	20	0.41	0.93	11.89
Littorina obtusata	31	0.06	0.81	3.22
Littorina plena	1	-	-	11.31
Littorina saxatilis	56	0.45	1.45	3.22
Littorina sitkana	19	0.24	0.93	8.55
Littorinimorpha sp. KL01	1	-	-	10.72
Littorinimorpha sp. KL03	1	-	-	8.26
Littorinimorpha sp. KL04	1	-	-	8.26
Loligo opalescens	3	0.06	0.17	21.11
Lottia alveus	1	-	-	22.56
Lottia digitalis	8	0.08	0.66	19.75
Lottia paradigitalis	14	0.11	0.51	18.12
Lottia pelta	6	0.41	0.79	18.12
Lottia scutum	8	0.38	1.48	23.45
Macoma balthica	23	0.45	3.65	15.46
Macoma calcarea	3	0.57	0.62	17.32
Macoma moesta	5	0.64	1.01	17.32
Macoma nasuta	1	-	-	15.46
Mactromeris polynyma	1	-	-	32.01
Margarites costalis	4	0.38	0.77	15.69
Margarites groenlandicus	1	-	-	10.43
Margarites helicinus	11	0	0	10.86
Margarites olivaceus	3	1.55	2.71	10.43
Margarites pupillus	2	0.43	0.43	10.86
Margarites sp. KL01	1	-	-	15.69
Mastigoteuthis magna	1	-	-	19.29
Melanella thersites	1	-	-	24.90
Mercenaria mercenaria	8	0.28	0.76	25.63
Mitrella cf. tuberosa	1	-	-	8.68
Modiolus modiolus	1	-	-	38.40
Montereina lentiginosa	1	-	-	15.60
Montereina nobilis	2	0.46	0.46	15.60
Mopalia imporcata	1	-	-	13.43
Mopalia kennerleyi	9	1.18	1.75	13.12
Mopalia lignosa	6	1.38	2.66	12.01
Mopalia vespertina	1	-	-	12.01
Musculus discors	1	-	-	12.13
Musculus niger	3	0.11	0.17	12.13
Mya arenaria	11	0.25	1.13	18.13
Mya truncata	6	4.41	8.34	18.13
Mytilus californianus	3	0.53	0.79	17.09
Mytilus edulis	79	8.84	18.96	0
Mytilus trossulus	17	5.90	17.82	0
Neptunea ithia	1	-	-	13.05
Neptunea phoenicea	1	-	-	13.05
Neverita lewisii	2	0	0	14.53
Nucella canaliculata	6	0.16	0.49	7.21
Nucella emarginata	23	0.10	0.46	6.46

Nucella lamellosa	3	0.33	0.34	6.85
Nucella lapillus	22	0.04	0.31	6.46
Nucella ostrina	6	0.59	1.45	7.21
Nucula proxima	1	-	-	24.98
Nuculana pernula	3	0.62	0.92	1.58
Nuculana radiata	1	-	-	1.58
Ocinebrina lurida	1	-	-	9.60
Ocinebrina sclera	1	-	-	9.60
Octopus rubescens	1	-	-	7.47
Odostomia sp. KL01	1	-	-	21.48
Odostomia sp. KL02	1	-	-	21.48
Oenopota bicarinata	4	0.18	0.31	11.56
Oenopota sp. KL01	4	0.92	1.61	4.91
Oenopota sp. KL02	1	-	-	8.53
Oenopota sp. KL03	1	-	-	4.91
Oenopota sp. KL04	1	-	-	8.53
Olivella baetica	1	-	-	14.5
Onchidella borealis	26	0.57	2.99	10.48
Onchidella cf. carpenteri	1	-	-	10.48
Onchidoris bilamellata	4	0.39	0.62	16.60
Onchidoris muricata	1	-	-	16.60
Palio dubia	3	0.43	0.64	4.27
Petaloconchus compactus	5	0	0	29.03
Philine lima	3	0	0	23.21
Pododesmus macrochisma	6	0.06	0.28	45.11
Polyschides carolinensis	1	-	-	18.64
Pomaulax gibberosus	1	-	-	20.09
Portlandia arctica	3	0	0	38.89
Propebela fidicula	1	-	-	11.35
Propebela turricula	7	2.43	4.17	11.35
Pulsellum salishorum	2	0.44	0.44	31.82
Puncturella galeata	1	-	-	27.25
Retusa obtusa	4	0.96	1.56	20.16
Rhabdus rectius	4	11.47	22.45	14.99
Rossia glaucopis	1	-	-	2.83
Rossia pacifica	5	0.06	0.16	2.83
Rossia palpebrosa	1	-	-	11.70
Rostanga pulchra	2	1.55	1.55	19.06
Ruditapes philippinarum	1	-	-	25.63
Saxidomus gigantea	4	0.36	0.62	26.69
Scabrotrophon fabricii	1	-	-	9.66
Scabrotrophon maltzani	3	0.72	1.08	9.66
Serpulorbis sp. KL01	1	-	-	29.03
Serripes laperousii	2	0.30	0.30	21.05
Similipecten greenlandicus	10	0.07	0.50	46.87
Siphonodentalium lobatum	2	0	0	22.92
Spisula solidissima	4	0.15	0.31	31.79
Stenosemus albus	5	0.05	0.15	20.15
Tachyrhynchus erosus	3	0.08	0.25	23.12
Tachyrhynchus reticulatus	4	1.49	2.98	25.25
Tegula funebralis	4	0.41	0.62	13.12
Tegula pulligo	4	0.31	0.46	13.12
Tellina nuculoides	3	0	0	18.91
Thyasira gouldi	7	10.04	17.72	17.97
Tonicella insignis	1	-	-	10.92
Tonicella lineata	15	1.12	2.99	5.07

Tonicella marmorea	44	0.42	1.75	10.92
Tonicella rubra	1	-	-	3.80
Tonicella undocaerulea	4	1.30	1.60	5.07
Tresus capax	4	0.62	0.93	31.79
Trichotropis cancellata	19	0.82	1.87	7.82
Triopha catalinae	3	17.99	26.39	16.59
Urosalpinx cinerea	7	0	0	11.27
Yoldia hyperborea	6	0.17	0.51	35.49
Yoldia myalis	2	0	0	43.80
Yoldiella frigida	2	0.31	0.31	14.26
Yoldiella nana	2	0.15	0.15	14.26
