# Supplementary material for: Patterns of DNA Barcode Variation in Canadian Marine Molluscs
Source: PLoS One. 2014 Apr 17;9(4):e95003. doi: 10.1371/journal.pone.0095003 (PMC3990619; doi:10.1371/journal.pone.0095003)
Supplement: Table S2 — Species found at each locality in this study. This table excludes 23 GenBank specimens (Scaphopoda) that lack locality information but are known to occur in Canada. (PDF) [file pone.0095003.s002.pdf]

Supporting Information Table S2: Species found at each locality in this study. This table excludes 23 GenBank specimens (Scaphopoda) that lack locality information but are known to occur in Canada.

Species	Region (Province/State)
**Bivalvia**	Bamfield (British Columbia)
Clinocardium nuttallii	
Crassostrea gigas	
Cyclocardia crassidens	
Keenocardium californiense	
Macoma nasuta	
Mya arenaria	
Mytilus californianus	
Mytilus trossulus	
Ruditapes philippinarum	
Tresus capax	
**Gastropoda**	
Amphissa columbiana	
Amphissa versicolor	
Armina californica	
Cadlina luteomarginata	
Calliostoma ligatum	
Ceratostoma foliatum	
Cidarina cidaris	
Crepipatella lingulata	
Diaulula sandiegensis	
Homalopoma baculum	
Fissurellidea bimaculata	
Flabellina verrucosa	
Littorina sitkana	
Lottia digitalis	
Lottia paradigitalis	
Melanella thersites	
Montereina nobilis	
Nucella lamellosa	
Nucella ostrina	
Ocinebrina lurida	
Odostomia sp. KL02	
Onchidella borealis	
Onchidella cf. carpenteri	
Onchidoris bilamellata	
Petaloconchus compactus	
Pomaulax gibberosus	
Puncturella galeata	
Serpulorbis sp. KL01	
Tegula pulligo	
Tegula funebralis	
Trichotropis cancellata	
**Polyplacophora**	
Cyanoplax dentiens	
Katharina tunicata	
Lepidochitona flectens	
Lepidozona interstincta	

Lepidozona radians	
Mopalia imporcata	
Tonicella undocaerulea	
**Scaphopoda**	
Rhabdus rectius	
**Bivalvia**	Haida Gwaii (British Columbia)
Mytilus trossulus	
Pododesmus macrochisma	
**Cephalopoda**	
Enteroctopus dofleini	
Loligo opalescens	
Octopus rubescens	
Rossia pacifica	
**Gastropoda**	
Acmaea mitra	
Alia carinata	
Amphissa reticulata	
Armina californica	
Calliostoma canaliculatum	
Calliostoma ligatum	
Cidarina cidaris	
Crepidula williamsi	
Crepipatella lingulata	
Dirona albolineata	
Doris montereyensis	
Flabellina verrucosa	
Haminoea virescens	
Homalopoma luridum	
Janolus fuscus	
Lacuna vincta	
Lirabuccinum dirum	
Lirularia succincta	
Littorina plena	
Littorina sitkana	
Lottia digitalis	
Lottia paradigitalis	
Lottia pelta	
Lottia scutum	
Mitrella cf. tuberosa	
Montereina lentiginosa	
Neptunea ithia	
Neptunea phoenicea	
Nucella canaliculata	
Nucella lamellosa	
Ocinebrina sclera	
Onchidoris muricata	
Rostanga pulchra	
Tegula pulligo	
**Polyplacophora**	
Cyanoplax dentiens	
Lepidochitona flectens	

Lepidozona interstincta<br>*Lepidozona radians*<br>*Leptochiton rugatus*<br>*Mopalia kennerleyi*<br>*Mopalia lignosa*<br>*Mopalia vespertina*<br>*Tonicella lineata*	
**Bivalvia**<br>*Crassostrea gigas*	Nanaimo (British Columbia)
**Bivalvia**<br>*Mytilus californianus*<br>*Mytilus trossulus*<br><br>**Gastropoda**<br>*Lottia paradigitalis*<br>*Lottia scutum*<br>*Nucella ostrina*<br>*Tegula funebralis*	Pacific Rim National Park (British Columbia)
**Cephalopoda**<br>*Dosidicus gigas*	Tofino (British Columbia)
**Bivalvia**<br>*Astarte montagui*<br>*Bathyarca pectunculoides*<br>*Ciliatocardium ciliatum*<br>*Cuspidaria glacialis*<br>*Cuspidaria subtorta*<br>*Ennucula tenuis*<br>*Macoma moesta*<br>*Musculus discors*<br>*Musculus niger*<br>*Nuculana pernula*<br>*Similipecten greenlandicus*<br>*Yoldia hyperborea*<br>*Yoldia myalis*<br>*Yoldiella frigida*<br>*Yoldiella nana*<br><br>**Gastropoda**<br>*Boreotrophon truncatus*<br>*Buccinum senshumaruae*<br>*Cryptonatica affinis*<br>*Cylichna alba*<br>*Cylichna cf. gouldii*<br>*Margarites costalis*<br>*Oenopota sp. KL04*<br>*Tachyrhynchus reticulatus*	Beaufort Sea
**Bivalvia**<br>*Axinopsida serricata*<br>*Clinocardium nuttallii*<br>*Glycymeris septentrionalis*<br>*Keenocardium blandum*	Cook Inlet (Alaska)

Keenocardium californiense
Kellia suborbicularis
Leukoma staminea
Mactromeris polynyma
Mytilus trossulus
Pododesmus macrochisma
Saxidomus gigantea
Tellina nuculoides

Gastropoda
Acanthodoris nanaimoensis
Acanthodoris pilosa
Acmaea mitra
Aeolidia papillosa
Amphissa columbiana
Ariadnaria insignis
Boreotrophon cepula
Buccinum aleuticum
Calliostoma ligatum
Crepidula williamsi
Cryptobranchia concentrica
Cryptonatica affinis
Cryptonatica aleutica
Cryptonatica russa
Discurria insessa
Doris montereyensis
Eubranchus rupium
Flabellina cf. trophina
Flabellina verrucosa
Gastropteron pacificum
Lacuna sp. KL01
Lacuna vincta
Lirabuccinum dirum
Lirularia succincta
Littorina sitkana
Littorinimorpha sp. KL01
Littorinimorpha sp. KL03
Littorinimorpha sp. KL04
Lottia alveus
Lottia digitalis
Lottia pelta
Lottia scutum
Margarites groenlandicus
Margarites pupillus
Neverita lewisii
Nucella canaliculata
Nucella emarginata
Odostomia sp. KL01
Olivella baetica
Onchidella borealis
Propebela fidicula
Scabrotrophon maltzani
Trichotropis cancellata
Triopha catalinae

**Polyplacophora**	
Boreochiton beringensis	
Cryptochiton stelleri	
Cyanoplax fernaldi	
Katharina tunicata	
Leptochiton alascensis	
Mopalia kennerleyi	
Tonicella insignis	
Tonicella lineata	
**Bivalvia**	Baffin Bay (Greenland)
Astarte montagui	
Bathyarca pectunculoides	
**Bivalvia**	Cornwallis Island (Nunvaut)
Mya truncata	
**Gastropoda**	
Aeolidia sp. KL02	
**Polyplacophora**	
Stenosemus albus	
**Gastropoda**	Devon Island (Nunavut)
Dendronotus frondosus	
Flabellina salmonacea	
Margarites olivaceus	
**Bivalvia**	Igloolik (Nunavut)
Ennucula tenuis	
Macoma calcarea	
Mya truncata	
Nuculana radiata	
Portlandia arctica	
Serripes laperousii	
**Cephalopoda**	
Rossia glaucopis	
**Gastropoda**	
Buccinum hydrophanum	
Clione limacina	
Cylichna cf. gouldii	
Dendronotus frondosus	
Flabellina salmonacea	
Margarites helicinus	
Margarites sp. KL01	
Scabrotrophon fabricii	
**Polyplacophora**	
Tonicella marmorea	
**Bivalvia**	Resolute (Nunavut)
Mya truncata	
Serripes laperousii	

**Cephalopoda**<br>*Rossia palpebrosa*<br><br>**Gastropoda**<br>*Admete sp. KL01*<br>*Aeolidia sp. 2*<br>*Buccinum hydrophanum*<br>*Buccinum sp. KL01*<br>*Clione limacina*<br>*Dendronotus frondosus*<br>*Margarites olivaceus*<br>*Oenopota sp. KL01*<br>*Oenopota sp. KL03*<br>*Philine lima*<br>*Retusa obtusa*<br><br>**Polyplacophora**<br>*Stenosemus albus*<br>*Tonicella marmorea*	
**Bivalvia**<br>*Axinopsida orbiculata*<br>*Crenella faba*<br>*Ennucula tenuis*<br>*Macoma balthica*<br>*Mytilus edulis*<br>*Mytilus trossulus*<br><br>**Gastropoda**<br>*Admete viridula*<br>*Aeolidia sp. KL01*<br>*Diaphana minuta*<br>*Ecrobia truncata*<br>*Flabellina sp. KL01*<br>*Littorina saxatilis*<br>*Limacina helicina*<br>*Margarites costalis*<br>*Margarites helicinus*<br>*Oenopota bicarinata*<br>*Oenopota sp. KL02*<br>*Philine lima*<br>*Propebela turricula*<br>*Retusa obtusa*<br><br>**Polyplacophora**<br>*Stenosemus albus*<br>*Tonicella marmorea*	Churchill (Manitoba)
**Gastropoda**<br>*Palio dubia*<br>*Propebela turricula*<br>*Tachyrhynchus erosus*	Baie Ste-Marguerite (Quebec)
**Bivalvia**<br>*Astarte borealis*<br>*Cyclocardia borealis*	St. Andrews (New Brunswick)

Mercenaria mercenaria<br>*Modiolus modiolus*<br>*Mytilus edulis*<br>*Mytilus trossulus*<br>*Nucula proxima*<br>*Spisula solidissima*<br><br>**Gastropoda**<br>*Buccinum undatum*<br>*Colus stimpsoni*<br>*Crepidula fornicata*<br>*Cuthona columbiana*<br>*Dendronotus cf. robustus*<br>*Dendronotus frondosus*<br>*Euspira heros*<br>*Euspira pallida*<br>*Flabellina salmonacea*<br>*Lacuna vincta*<br>*Littorina littorea*<br>*Littorina obtusata*<br>*Littorina saxatilis*<br>*Nucella lapillus*<br>*Onchidoris bilamellata*<br><br>**Polyplacophora**<br>*Tonicella marmorea*<br>*Tonicella rubra*	
**Gastropoda**<br>*Littorina littorea*	Fundy National Park (New Brunswick)
**Cephalopoda**<br>*Brachioteuthis beani*<br>*Mastigoteuthis magna*	Maritimes Region
**Bivalvia**<br>*Mytilus edulis*<br>*Mytilus trossulus*<br><br>**Gastropoda**<br>*Lacuna vincta*<br>*Littorina littorea*<br>*Littorina obtusata*<br>*Littorina saxatilis*<br>*Nucella lapillus*	Peggy’s Cove (Nova Scotia)
**Bivalvia**<br>*Mytilus edulis*<br><br>**Gastropoda**<br>*Littorina obtusata*<br>*Littorina saxatilis*<br>*Urosalpinx cinerea*	Lunenburg (Nova Scotia)
**Bivalvia**<br>*Mytilus edulis*	Prospect (Nova Scotia)

**Gastropoda**<br>*Littorina obtusata*<br>*Littorina littorea*	
**Bivalvia**<br>*Gemma gemma*<br><br>**Gastropoda**<br>*Euspira heros*<br>*Littorina littorea*	Riverport (Nova Scotia)
**Gastropoda**<br>*Ilyanassa obsoleta*	Wolfville (Nova Scotia)
**Gastropoda**<br>*Ilyanassa obsoleta*<br>*Littorina saxatilis*	Cavendish (Prince Edward Island)
**Bivalvia**<br>*Gemma gemma*<br>*Mercenaria mercenaria*<br><br>**Gastropoda**<br>*Boonea cf. bisuturalis*<br>*Ecrobia truncata*<br>*Ilyanassa obsoleta*<br>*Littorina littorea*<br>*Littorina saxatilis*	Malpeque Bay (Prince Edward Island)
**Bivalvia**<br>*Crassostrea virginica*<br>*Mercenaria mercenaria*	Summerside (Prince Edward Island)
**Bivalvia**<br>*Crassostrea virginica*<br>*Ensis directus*<br>*Mytilus edulis*<br><br>**Gastropoda**<br>*Ilyanassa obsoleta*<br>*Nucella lapillus*	Tea Hill (Prince Edward Island)
**Bivalvia**<br>*Macoma balthica*<br>*Mya arenaria*<br>*Thyasira gouldi*	Bonne Bay (Newfoundland and Labrador)
**Bivalvia**<br>*Mya arenaria*	Main Brook (Newfoundland and Labrador)
**Bivalvia**<br>*Ennucula tenuis*	Nachvak Fjord (Newfoundland and Labrador)

**Polyplacophora**<br>*Tonicella marmorea*	
**Bivalvia**<br>*Yoldia hyperborea*	Saglek Fjord (Newfoundland and Labrador)
**Gastropoda**<br>*Littorina littorea*<br>*Nucella lapillus*	Terra Nova National Park (Newfoundland and Labrador)
**Scaphopoda**<br>*Siphonodentalium lobatum*	Iceland (Northwest Atlantic)
